# Femtosecond Laser Precision Etching of Silver Layer on Silica Aerogel Surfaces

**DOI:** 10.3390/mi16101107

**Published:** 2025-09-29

**Authors:** Shengtian Lin, Congyi Wu, Guojun Zhang, Jinjin Wu

**Affiliations:** Analytical and Testing Center of Huazhong University of Science and Technology, Huazhong University of Science and Technology, No. 1037, Luoyu Road, Wuhan 430074, Chinawucongyi@hust.edu.cn (C.W.); zgj@hust.edu.cn (G.Z.)

**Keywords:** femtosecond laser, silica aerogel, Ag film, laser etching

## Abstract

Silica fiber-reinforced silica aerogel (SFRSA) has low dielectric constant, light weight and high temperature resistance characteristics, making it one of the preferred materials for heat-resistant absorptive layers on the surfaces of high-speed aircraft. However, due to its ultra-high porosity, poor rigidity, and sensitivity to organic solvents, existing machining and chemical etching processes struggle to achieve patterned preparation of metallic layers on aerogel substrates. In order to address this issue, the present study employs femtosecond laser etching of the metal layer on the SFRSA surface. Orthogonal experiments were conducted to analyze the impact of different laser process parameters on the etching quality. With straightness as the primary factor, the optimal process parameters obtained were a laser power set to 2.15 W, a laser etching speed of 200 mm/s, and a laser etching time of 9. This achieved an etching width of 26.16 μm, a heat-affected zone of 39.16 μm, and straightness of 7.9 μm. Finally, Raman spectroscopy was used to study laser-ablated samples; thermogravimetric analysis (TGA) and Pyrolysis-Gas Chromatography–Mass Spectrometry analysis (Py-GC-MS) were employed to investigate the changes in the metal layer at high temperatures. A compositional analysis was conducted, revealing a decrease in carbon content within the etched region following laser ablation. The production of CO_2_ gas and surface oxidation indicated that laser etching primarily operates via a photothermal mechanism.

## 1. Introduction

The material under consideration in this study is SiO_2_ fiber-reinforced SiO_2_ aerogel (SFRSA), which possesses the following characteristics: low dielectric constant [1], lightweight [2], and high-temperature resistance [3]. This study found that these characteristics make SFRSA one of the preferred materials for current aerospace applications [4]. The arrangement of metal unit arrays on this material enables the selective transmission or reflection of electromagnetic waves [5], which is a process that has found widespread application in the aerospace field [6]. The process of first depositing a metal layer and then patterning it is a viable method for preparing high-resolution patterns. Currently, patterns with line widths and line spacings both less than 50 μm have been achieved on PET, silicon wafers, and glass substrates. However, due to the ultra-high porosity (>80% [7,8,9]) of aerogel surfaces, they tend to be uneven and have low mechanical strength. The method of first depositing a metal layer and then patterning it faces challenges such as low patterning resolution and significant damage to the aerogel substrate [10,11,12,13].

Laser etching offers advantages such as precision [14], flexibility, and efficiency [15]. Different materials exhibit varying energy absorption characteristics at different laser wavelengths, which directly impacts etching quality. Infrared lasers can rapidly heat the surface of metallic silver above its melting point, causing melting and evaporation to remove the material [16]. However, this process exhibits significant thermal effects [17]. Ultraviolet lasers have shorter wavelengths and higher photon energy, enabling direct breaking of silver’s metallic bonds (without thermal conduction), with material detaching from the surface in atomic/molecular form, resulting in minimal thermal diffusion. Greater etching depth can enhance etching efficiency [18]. Laser pulse width is also a critical parameter in the etching process, directly affecting the size of the heat-affected zone [19,20], processing accuracy [21], and etching quality [22]. Compared to nanosecond lasers [23] and picosecond lasers [24], femtosecond lasers [25] have shorter pulse widths, enabling cold etching with minimal thermal damage to the material surface. Currently, attempts have been made to use femtosecond lasers to etch Ag layers on glass-based [26] and PET polymer [27] substrates. However, the process rules for etching Ag coatings on aerogel substrates are still incomplete; additionally, the interaction mechanism between the laser and the silver coating remains unclear. Therefore, there is a lack of systematic research on femtosecond ultraviolet laser etching of Ag coatings on aerogel substrates.

In this study, silver coatings were prepared on the surface of aerogel substrates using screen printing and sintering. The utilization of a femtosecond ultraviolet laser to etch the silver coatings on the aerogel substrates yielded high-quality etching results. Firstly, an evaluation system for etching quality was established. This system was based on etching width, the heat-affected zone size, and straightness. These were analyzed by studying the etched surface morphology. Second, a three-factor, five-level orthogonal experiment was designed to analyze the influence of laser etching parameters on the etched surface morphology. Subsequently, the effect of a single factor—etching frequency—on cross-sectional morphology was examined. Interaction analysis was conducted to investigate the mutual influence among the factors. Finally, the laser etching mechanism was elucidated through combined analysis of Raman spectroscopy, EDS, TGA, and Py-GC-MS. The present work provides references for manufacturing technology and mechanistic support for the etching of silver coatings on aerogel substrates.

## 2. Experiments

### 2.1. Materials

Ethyl Cellulose (Analytically Pure AR) was purchased from Tianjin Komeo Chemical Reagent Co. (Tianjin, China). Hydrogenated Castor Oil, Triethyl Citrate, and Silver Powder were purchased from Shanghai McLean Biochemical Technology Co. (Shanghai, China). Diethylene Glycol Monobutyl Ether (Analytical Pure AR) and Pinoresinol (Analytical Pure AR) were provided by Xilong Chemical Company (Shantou, China); Trillatone X-100 (Chemically Pure CP) was provided by Xilong Science Co. (Shantou, China). SiO_2_ Fiber-Reinforced Phase Aerogel was provided by Hunan Ronglan Intelligent Technology Co. (Xiangtan, China). Glass Powder was supplied by Hunyuan Junhong New Material Co. (Datong, China).

### 2.2. Aerogel-Based Silver Coating Preparation

First, the organic carrier was prepared by mixing the organic solvent and stirring it thoroughly. Then, the silver powder, glass powder and organic carrier were stirred together for 10 min. The silver paste was then printed onto the aerogel surface using a 200 mesh screen. Following a drying process at ambient temperature, the printed samples were subjected to sintering in an air atmosphere. During the process of sintering, the glass phase underwent melting, thereby forming a bonding phase that consolidated the silver particles. The samples were heated at a rate of 5 °C/min to 765 °C, held at this temperature for 10 min, and then cooled with the furnace. The thickness of the sintered silver layer is measured to range from 40 to 60 μm.

### 2.3. Laser Ablation System

**Figure 1:**
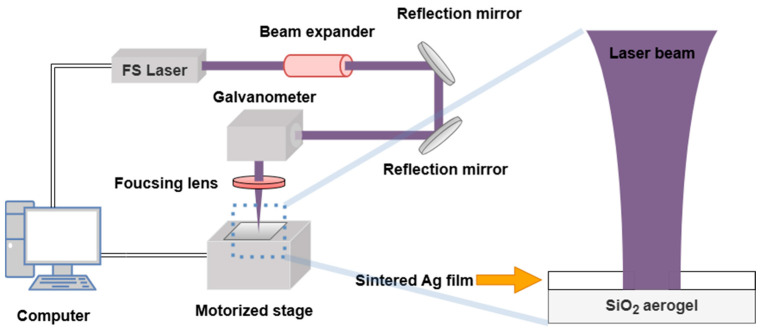
Schematic illustration of the laser ablation system.

As demonstrated in Figure 1, the laser ablation system utilized in this study is predominantly composed of two primary components: an optical path segment and a control element. The optical path part principally consists of a femtosecond ultraviolet laser, a beam expander mirror, a reflector, a galvanometer, and a focusing lens. The femtosecond UV laser (Tangerine, Amplitude Systems, Pessac, France) utilized in this study possesses an output pulse width of 150 fs@200 kHz, a light output diameter of 4.2 mm, a beam diameter of less than 20 μm, and a repetition frequency (RR) ranging from 100 kHz to 1 MHz. A 4-fold beam expander (MOTEX-Motorized Variable Beam Expander, LINOS, Wisbaden, Germany) was employed to enhance beam collimation. The focusing lens (FP0884, Jenoptik, Jena, Germany) had a focal length of 103 mm, and the moving stage was located on the focal plane. The ablation experiments were conducted under ambient air conditions, without the use of auxiliary gases. The control section primarily comprised an industrial personal computer (IPC) and the motion stage. The base of the aerogel, which was composed of sintered silver-coated material, was then placed on the motion stage. Subsequent to laser ablation, the ablated area residue was blown sequentially with an air gun, and the sample ablated area was cleaned with an ethanol solution before being dried at room temperature.

### 2.4. Design of Experiments

Preliminary exploratory experiments have indicated that laser power (LP), laser etching speed (LES) and laser etching times (LET) are the key factors in laser removal of coatings. Preliminary test results indicate that when the LP, LES, and LET are in the range of 1.6–3.8 W, 50–250 mm/s, and 1–9 times, respectively, the quality of laser etching is enhanced, and there is no occurrence of material that cannot be removed or is severely etched. An experiment was designed with three factors and five levels, following an orthogonal design. The aim of the experiment was to evaluate the effect of combinations of process parameters on the quality of laser-removed coatings. The levels and specific values of these process parameters are shown in Table 1, and the specific process parameters in each experiment are shown in Table 2.

### 2.5. Characterization

The surface morphology of laser ablation was observed using an optical confocal microscope (VHX-E20, KEYENCE, Osaka, Japan). The cross-sectional topography of the ablated coating and the distribution of elements on the surface of the ablated area were obtained using a scanning electron microscope (SU3900, HITACHI, Tokyo, Japan). The Raman spectra of the samples were recorded using a laser confocal micro-Raman spectrometer from HORIBA Jobin Yvon, Paris, France, equipped with a 30 mW helium–cadmium laser (532 nm) with a Raman shift range of 100–4000 cm^−1^ at room temperature. Thermogravimetric Infrared Correlator (TGA-IR, PerkinElmer, Hopkinton, MA, USA) was utilized for the purpose of thermogravimetric analysis (TGA) of the samples, as well as the collection of infrared spectra of the produced gases during the heating process (air conditions, ramping up to 800 °C at a rate of 10 °C/min).

## 3. Result and Discussion

### 3.1. Evaluation of Laser Seam

**Figure 2:**
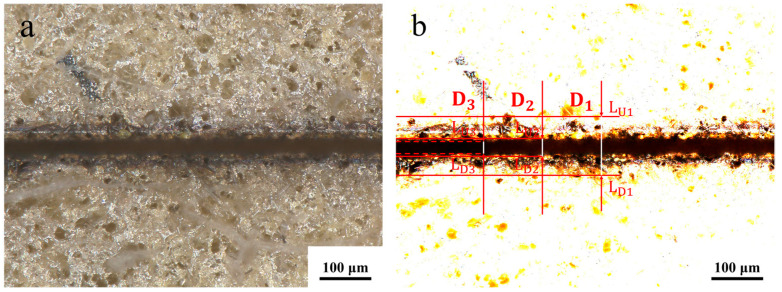
Schematic diagram of the laser ablation quality evaluation. (**a**) The original surface morphology and (**b**) contrast-enhanced image of specimen 14 following laser etching are illustrated.

The laser etching quality evaluation system was established based on the surface morphology of the etched seam, as illustrated in Figure 2. Evidence indicates the presence of a region that differs from the initial coating surface on both sides of the seam edge, which is known as the heat-affected zone. The upper and lower lines, designated L_U1_ and L_U2_, respectively, are drawn along the perimeter of the heat-affected zone. The seam edge is not a perfect straight line, and the upper tangent L_U2_ and lower tangent L_D3_ can be drawn along the upper edge of the seam. Similarly, the upper tangent L_D2_ and lower tangent L_D3_ can be drawn along the lower edge of the seam. The spacing between L_U1_ and L_D1_ is D_1_, the spacing between L_U2_ and L_D2_ is D_2_, and the spacing between L_U3_ and L_D3_ is D_3_. The determination of the extent to which the coating has been removed in the ablated area is contingent upon the analysis of the seam surface morphology in conjunction with the seam cross-section. It is evident that, in accordance with the values of D_1_, D_2_ and D_3_, the evaluation indexes of the width of the heat-affected zone (W_H_), the width of the ablation (W_S_), and the straightness of the seam edge (S_E_) can be determined. The following formulas have been deduced for these evaluation indices:

(1) $$W_{H}=\sum_{j=1}^{5}\left(D_{1\left(j\right)}-D_{2\left(j\right)}\right)/10$$

(2) $$W_{S}=\sum_{j=1}^{5}D_{2\left(j\right)}/5$$

(3) $$S_{E}=\sum_{j=1}^{5}\left(D_{2\left(j\right)}-D_{3\left(j\right)}\right)/10$$

**Figure 3:**
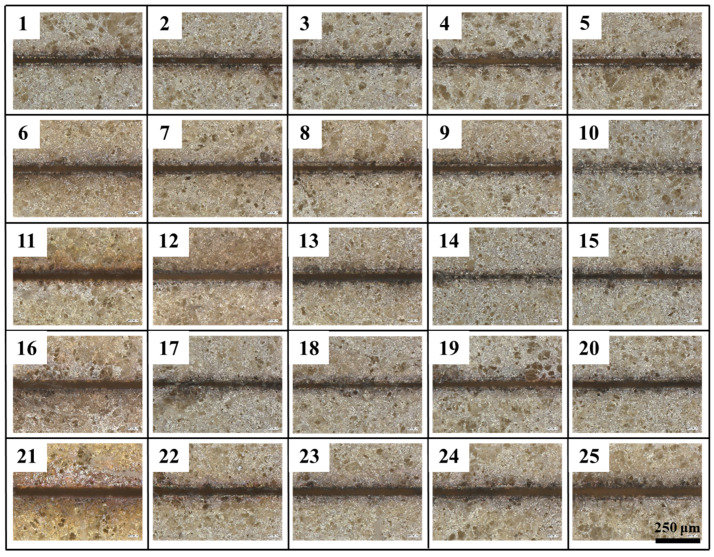
Optical micrographs of the samples at 200×.

Since straightness reflects edge etching precision, it is the primary consideration in the etching evaluation system, followed by the heat-affected zone and etching width. Meanwhile, whether the coating has been completely etched away is determined by the absence of residual coating within the etching seams. It is important to note that five samples with identical parameters were selected for the calculation of the mean value for the evaluation indexes under differing process parameters. The surface morphology of each etching process is illustrated in Figure 3, and the statistical data are presented in Table 3.

### 3.2. Orthogonal Experiment Analysis of Variance

Extreme variance analysis and analysis of variance (ANOVA) are utilized in orthogonal tests to evaluate the impact of multiple factors on the outcomes of experiments. Polar deviation (R) is defined as the difference between the maximum and minimum values in the data set, with the value serving to indicate the range of fluctuation in the data. The key variables affecting the etching effect can be expeditiously screened by means of extreme variance analysis, thus facilitating the determination of the optimal combination of levels under differing etching evaluation indexes. Analysis of variance (ANOVA) is a statistical method that aims to determine the influence of controllable factors on the results of a study by examining the contribution of different sources of variation to the total variation. As demonstrated in the accompanying Table 4, Table 5 and Table 6, the analysis of variance (ANOVA) encompasses a series of statistical components, including the sum of squares (SS), degrees of freedom (DOF), mean square (MS), F-value, *p*-value, and the extent to which these elements contribute to the outcomes of the study. As illustrated in Figure 3, the etched area morphology exhibited variability in its response to each test. Specifically, test10, 14, 15, 17 and 18 failed to achieve complete removal of the coating, resulting in residue formation within the etched area.

**Figure 4:**
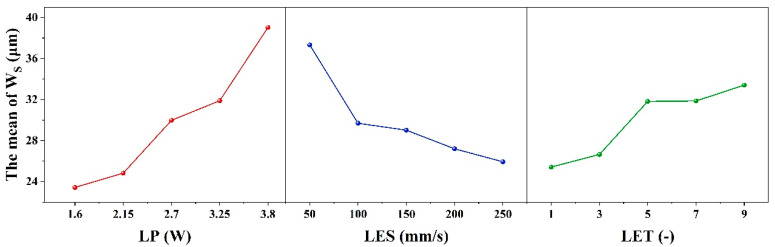
Factors Affecting the width of the seam: Laser Power, Laser etching speed, Laser etching times.

When the evaluation index is set to W_S_, the results of the range analysis for each process parameter are shown in Table 7 and Figure 4. The effect of LET on the dependent variable exhibits a nonlinear threshold trend, first increasing and then leveling off: it is significant at low levels but not at high levels, with the high-low effects canceling each other out to result in an overall non-significant effect.

The range R_1_ of the process parameter LP is 15.59 μm, and the optimum level is 1 (1.6 W). The range R_2_ for the process parameter of LES is 11.39 μm, with an optimum level of 5 (250 mm/s). The process parameter LET is defined as follows: R_3_ ranges from 7.99 μm with an optimal value of 1 (1 times). Therefore, the minimum W_S_ is theoretically obtained when the combinations of ablation process parameters are as follows: 1 (1.6 W), 5 (250 mm/s), and 1 (1 times). However, according to the combination of process parameters in the orthogonal table, it is evident that this combination of process parameters is not capable of removing the coating. Furthermore, the order of magnitude of the contribution share of each factor in Table 4 is consistent with the order of magnitude of the extreme deviation (R_j_) in Table 7, thereby indicating that LP, LES and LET have a decreasing effect on W_S_. It was determined that the conditions for complete removal of the coating from the etched area were met when the following parameter combination was utilized: 1 (1.6 W)-5 (250 mm/s)-5 (9 times) with a minimum W_S_ of 21.05 μm. This determination was made based on the orthogonal table ranges.

**Figure 5:**
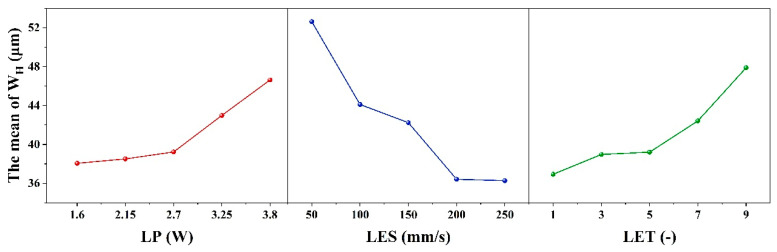
Factors Affecting the heat-affected zone: Laser Power, Laser etching speed, and Laser etching times.

Table 8 and Figure 5 show the results of the range analysis for each process parameter when the etching evaluation index is W_H_. The effect of LP on the dependent variable exhibits a nonlinear threshold trend, initially stable before increasing. It is insignificant at low levels but significant at high levels, with the high-low effects canceling each other out to yield overall insignificance. For LP, R_1_ is 8.56 μm and the optimum level is 1 (1.6 W); for LES, R_2_ is 13.89 μm and the optimum level is 5 (250 mm/s); and for LET, R_3_ is 10.96 μm and the optimum level is 1 (1 time). The minimum W_H_ is therefore obtained with the combination of etching process parameters 1 (1.6 W)-5 (250 mm/s)-1 (1 times). However, according to the combination of process parameters 2 (2.15 W)-5 (250 mm/s)-1 (1 times) in the orthogonal table, this process combination cannot remove the coating. Additionally, the contribution share of each factor in Table 5 is consistent with the extreme deviation (R_j_) in Table 8, indicating that LES, LET and LP decrease W_H_. Based on the ranges in the orthogonal table, it was determined that the coating was completely removed from the etched area when the parameter combination was 1 (1.6 W)-1 (50 mm/s)-1 (1 times), with a minimum W_H_ of 36.85 μm.

**Figure 6:**
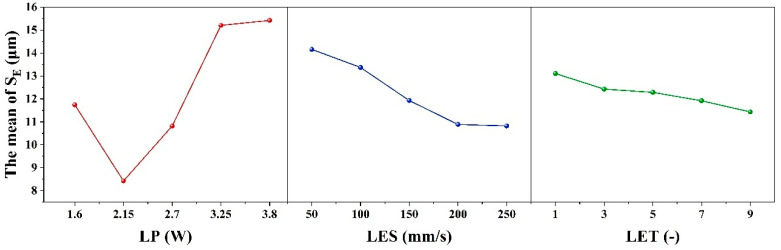
Factors Affecting the straightness of the seam: Laser Power, Laser etching speed, and Laser etching times.

As illustrated in Table 9 and Figure 6, the results of the range analysis for each process parameter are presented, with the etching evaluation index designated as S_E_. The excessively strong effect of LP on the dependent variable makes the effects of LES and LET appear relatively weak, leading to an overall lack of significance in the model. The range of the process parameter LP is 7.16 μm, with an optimum level of 2 (2.15 W). For the process parameter LES, the range is 1.68 μm, and the optimum level is 5 (250 mm/s). Finally, for the process parameter LET, the range is 1.68 μm, and the optimum level is 5 (9 times). The order of magnitude of the contribution shares of the factors in Table 6 and the order of magnitude of the extreme deviation (R_j_) in Table 9 are consistent, indicating that LP, LES and LET have a decreasing effect on S_E_. The condition of complete removal of the coating from the etched area is satisfied when the parameter combination is 2 (2.15 W)-4 (200 mm/s)-5 (9 times) and the minimum S_E_ is 7.9 μm, according to the range of the orthogonal table. Therefore, the minimum S_E_ is obtained when the parameter combination of the etching process is 2 (2.15 W)-4 (200 mm/s)-5 (9 times).

### 3.3. Interaction Analysis

**Figure 7:**
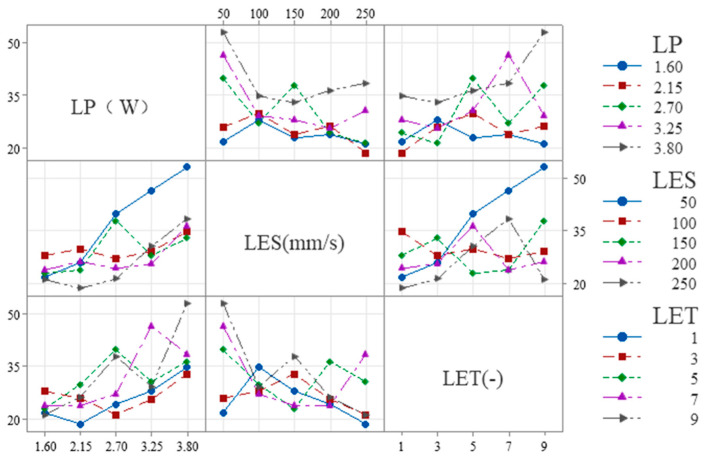
Interactions between each process parameter in W_S_.

Interaction analysis can rapidly determine correlations between factors under the same evaluation metric. The notation A*B denotes the interaction between laser process parameters A and B. Figure 5, Figure 6 and Figure 7 illustrate the correlations among process parameters when the evaluation metrics are LP, LES, and LET. The results are as follows:

The following is shown in Figure 7 for W_S_:

(1) LES × LP: The slope is steeper at low LES (50–100 mm/s) and flatter at high LES (200–250 mm/s), indicating that overall, the influence of LES on LP diminishes as LES increases.

(2) LET × LP: The slope is smaller at low LET (1–3) and larger at high LET (7–9), indicating that overall, as LET increases, its influence on LP tends to increase.

(3) LET × LES: The slope is smaller at low LET (1–3) and larger at high LET (7–9), indicating that overall, as LET increases, its influence on LES tends to increase.

**Figure 8:**
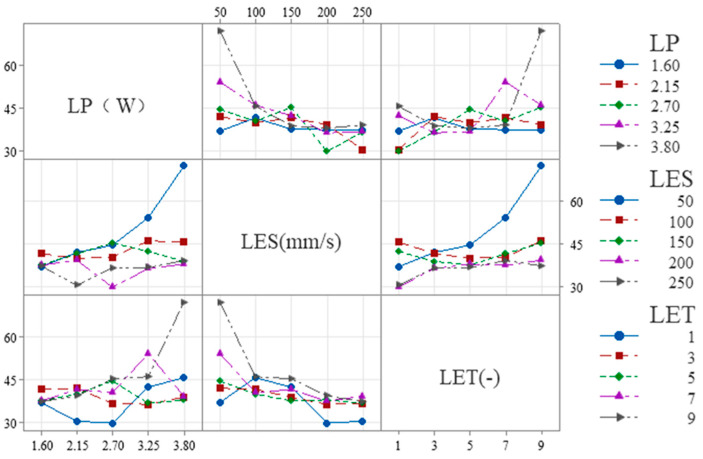
Interactions between each process parameter in W_H_.

The following is shown in Figure 8 for W_H_:

(1) LES × LP: The slope is steeper at low LES (50–100 mm/s) and flatter at high LES (200–250 mm/s), indicating that overall, the influence of LES on LP diminishes as LES increases.

(2) LET × LP: The slope is smaller at low LET (1–3) and larger at high LET (7–9), indicating that overall, as LET increases, its influence on LP tends to increase.

(3) LET × LES: The slope is smaller at low LET (1–3) and larger at high LET (7–9), indicating that overall, as LET increases, its influence on LES tends to increase.

**Figure 9:**
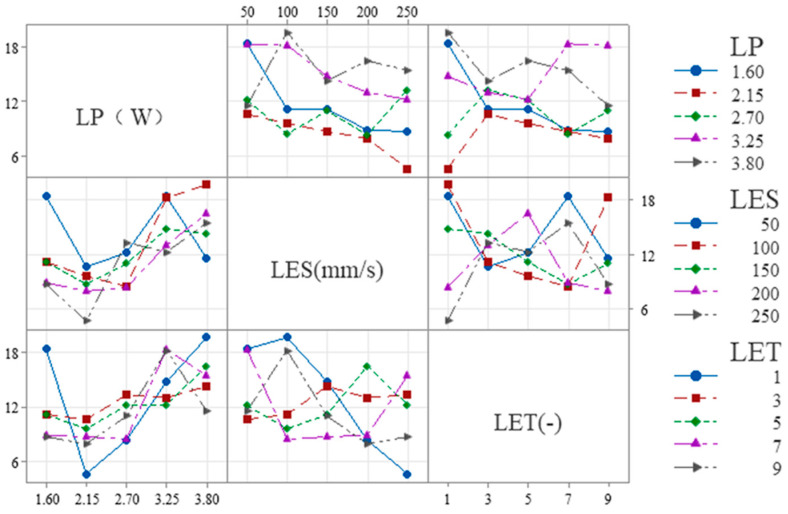
Interactions between each process parameter in S_E_.

The following is shown in Figure 9 for S_E_:

(1) LES × LP: The slope is steeper at low LES (50–100 mm/s) and flatter at high LES (200–250 mm/s), indicating that overall, the influence of LES on LP diminishes as LES increases.

(2) LET × LP: The slope is steeper at low LET (1–3) and larger at high LET (7–9), indicating that overall, as LET increases, its influence on LP tends to increase.

(3) LET × LES: The slope at low LET (1–3) is similar to that at high LET (7–9), both being higher than the slope at medium LET (3–7). This indicates that, overall, the effect of LET on LES first decreases and then increases as LET increases.

### 3.4. Effect of Etching Times on Etching Cross-Section

**Figure 10:**
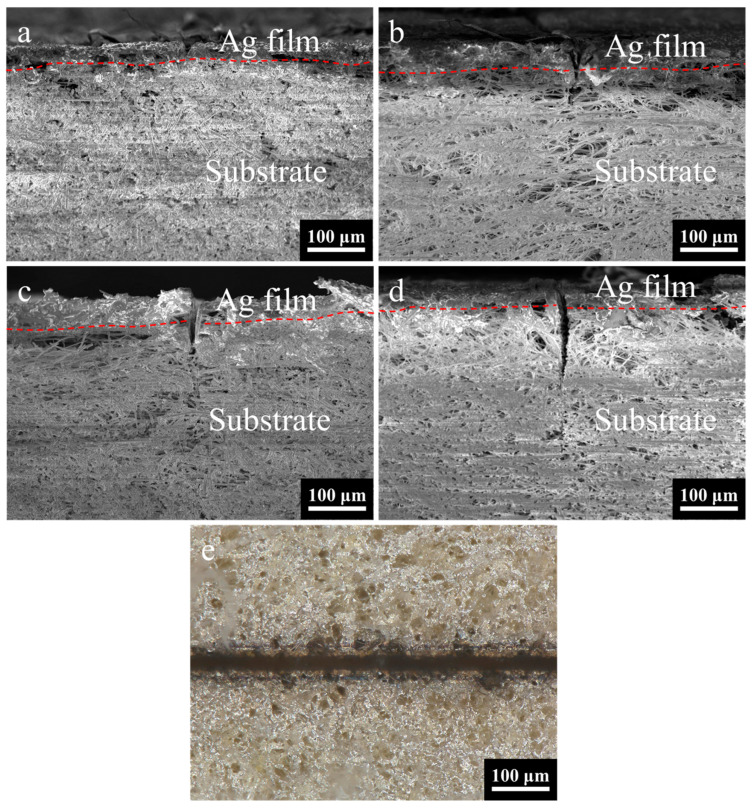
(**a**–**d**) SEM images of the cross-section of silver coating on aerogel substrate under different etching times. (**e**) Optical image after etching of the optimal parameters of the silver coating based on aerogel. The red dotted line in the figure serves as the boundary between the silver film and the substrate.

The number of laser etching times is found to be a crucial factor in achieving complete removal of the coating. The optimal process parameters are determined through experimentation and analysis. As illustrated in Figure 10a, the cross-sectional morphology is observed under the condition of 7 etching times. The energy injected into the surface by the laser is rapidly carried away by the removed material before it can diffuse, resulting in a remarkably smooth etched edge on the silver layer. In this instance, the etching depth was measured at 21.08 μm.

As illustrated in Figure 10b–d, there is a demonstrable change in etching depth when the number of etching times is changed from 9 to 13. The etching depth increases from 89.72 μm to 143.29 μm. It has been demonstrated that the etching depth increases in proportion to etching times. It is noteworthy that when etching times are set at 9, the result is the complete removal of the coating. It is evident that an increase in the etching times results in an increase in etching width and greater substrate damage. This observation indicates that 9 times are sufficient to completely remove the coating, with the optimal parameters being 2.15 W-200 mm/s-9 times.

The optical image of the etched surface morphology is shown in Figure 10e, captured under the optimal process parameter combination of 2.15 W-200 mm/s-9 times, and the mean etching width is measured at 26.16 μm, the mean width of the heat-affected zone is recorded at 39.16 μm, and the mean straightness width is determined at 7.9 μm (based on five samples). As demonstrated in Figure 10b, the etching edges are characterized by a smooth finish. This finding suggests that, within the specified process parameter configuration, the requirements for precise etching with minimal damage can be fulfilled.

### 3.5. Mechanism of the Femtosecond UV Laser Ablation

**Figure 11:**
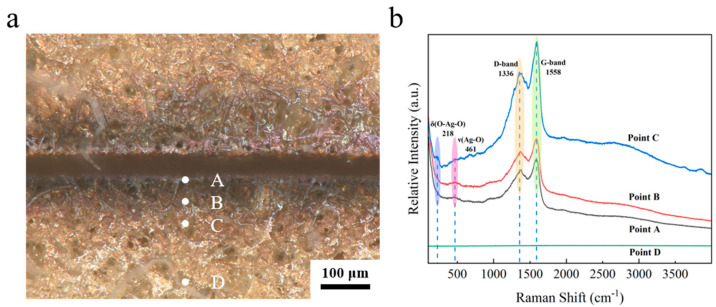
(**a**) Optical micrographs of the seam and (**b**) Raman measurements in different points.

Figure 11a clearly shows that the color of the edge of the seam is blackened. In order to investigate the mechanism of etching of the ultraviolet femtosecond laser, a compositional analysis was conducted of the slit edges. As demonstrated in Figure 11a, the local morphology of silver layer etching involves the selection of four points, A, B, C and D, which are positioned from the interior to the exterior. These points correspond to the inner wall of the etching edge, the etching edge itself, the heat-affected zone, and the unaffected zone, respectively. As demonstrated in Figure 11b, the Raman results indicate the presence of broad superposition peaks in the range of 1351–1595 cm^−1^ at points A, B, and C, which are attributed to the D-band (defect-induced peaks) and the G-band (graphitization peaks) of the amorphous carbon [28]. The elements present in the amorphous carbon and graphite may originate from the organic vehicle in the silver paste. During the sintering process, the organic vehicle did not volatilize completely, resulting in the residual organic components in the sintered silver layer [29]. The broad peaks observed at both points A and B near 461 cm^−1^ can be attributed to the Ag-O bond stretching vibrations [30,31]. As demonstrated in Figure 11b, the peaks at Raman shifts of 218 cm^−1^ and 461 cm^−1^ at point C correspond to the bending vibration of O-Ag-O and the stretching vibration of the Ag-O bond [32], respectively. Compared to points A and B, the spectral line corresponding to point C exhibits a stronger peak. This is because points A and B are closer to the etching center, where the amorphous carbon generated is directly oxidized and removed due to excessively high temperatures. In contrast, point C is farther from the etching center, where temperatures are relatively lower, allowing most of the generated amorphous carbon to deposit in this region. Point D is even farther from the etching zone, lacking sufficient laser energy input. Moreover, the sintered silver layer’s surface is primarily composed of silver, whose spectral line appears as a flat line. Consequently, point D exhibits a flat line with no peaks. The results of the Raman test conducted on the ablated area indicate the presence of amorphous carbon in the vicinity, along with signs of oxidation within the ablated area itself.

**Figure 12:**
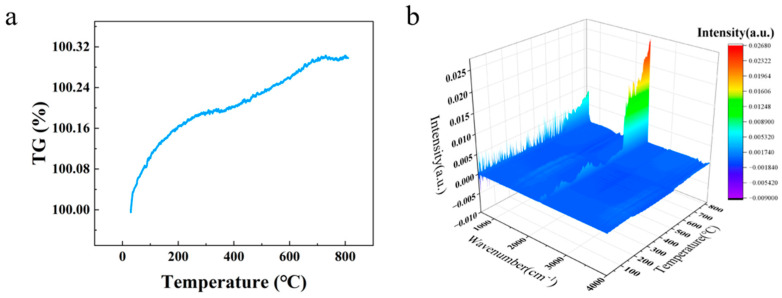
(**a**) TG curve of the sintered Ag film and (**b**) 3D-FTIR spectra of volatile products during heating of sintered Ag film.

Furthermore, in order to facilitate a more profound analysis of the thermophysical change process of the composites under femtosecond laser etching, thermogravimetric tests and FTIR spectroscopy of gas products at differing temperatures were performed on the matrix and coating of the composites, respectively. As demonstrated in Figure 12a, the quality of the silver coating exhibited stability within the temperature range of room temperature to 800 °C, as evidenced by the TG curves. The curve demonstrates a marginal overall increase from 100% to 100.3%. In accordance with the established formula, 2Ag + 1/2 O_2_ = Ag_2_O, it can be deduced that the mass of silver undergoes an approximate 10.7% increase following oxidation. However, the actual increase in mass is negligible, suggesting that only the surface layer is oxidized, resulting in a marginal increase in mass. As shown in Figure 12b, no identifiable infrared peaks appear below 650 °C. When the temperature exceeds 650 °C, the characteristic peak for the asymmetric stretching vibration of CO_2_ appears around 2300 cm^−1^ [33]. This phenomenon is attributable to the decomposition of residual carbon within the coating, which results in the generation of CO_2_.

**Figure 13:**
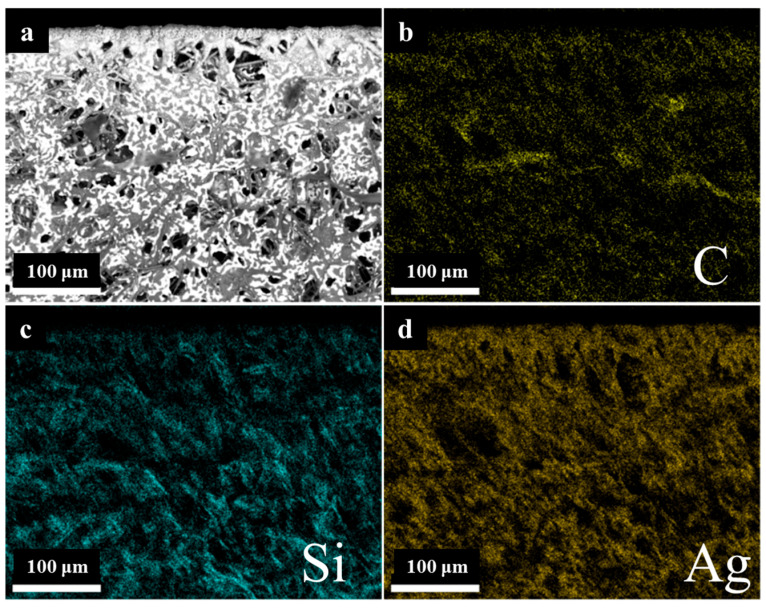
SEM-EDAX around the seam. (**a**) SEM image of the seam; (**b**) C; (**c**) Si and (**d**) Ag element distribution around the seam.

Furthermore, the elemental distribution surrounding the etched area was analyzed by means of SEM energy dispersive X-ray analysis (SEM-EDAX), as illustrated in Figure 13. The elemental distribution of Si and Ag does not differ significantly from that of the initial surface, whereas the distribution of C in the vicinity of the etched area is low, presumably due to the rapid oxidation of C to CO_2_ under the influence of high laser energy. It can be posited that the elemental distribution of C is principally located within the heat-affected zone. This observation suggests that etching spatters are more frequently deposited in this area.

**Figure 14:**
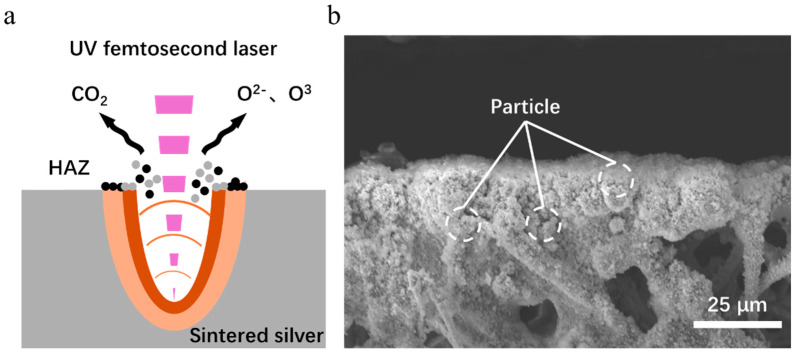
(**a**) Schematic of interaction mechanism between the femtosecond UV laser and the sintered silver; (**b**) SEM image of particles deposited at the seam edge.

As shown in Figure 14a, the ablation mechanism of the material primarily manifests as photothermal ablation [34]. During laser–material interaction, the high-energy laser ionizes the air to produce reactive oxygen species (atomic oxygen and ozone), while the laser-induced high-temperature, high-pressure plasma (electron density > 10^17^ cm^−3^, temperature > 5000 K) [35] ejects a large amount of gaseous material (Ag, C). The reactive oxygen species (Ag, C) then evaporate. The gaseous substances (Ag, C) that are splashed out react with the reactive oxygen species, resulting in the oxidation of gaseous silver by cooling and its deposition around the seam, as shown in Figure 14b. Part of the vaporized carbon impurities are oxidized and converted to CO_2_, which dissipates in the air; the other part is deposited in the heat-affected zone.

## 4. Conclusions

This study first defined a laser ablation quality evaluation system based on W_S_, W_H_, and S_E_ using femtosecond ultraviolet laser etching. Next, orthogonal experiments were conducted to analyze the impact of laser etching parameters on etching quality. Finally, the interaction mechanism between femtosecond ultraviolet lasers and sintered silver layers was investigated.

The conclusions are as follows:

Within the scope of the orthogonal experiments in this study, considering the W_S_, W_H_, S_E_ size, and whether the coating was completely removed, the optimal process parameter combination was 2 (2.15 W)-5 (250 mm/s)-5 (9 times). Under this parameter combination, an etching width of 26.16 μm, a heat-affected zone of 39.16 μm, and a straightness of 7.9 μm were achieved.

In the orthogonal experiments of this study, changes in W_S_ and W_H_ followed similar patterns, both increasing with increases in LW and LET and decreases in LES. S_E_ decreases with increases in LES and LET, and first decreases and then increases with increases in LP.

During the interaction between the femtosecond ultraviolet laser and the sintered silver layer, the sintered silver coating was removed through photothermal ablation, and it was confirmed that silver oxide particles and carbonized residues remained near the seam. Furthermore, during the ablation process, no toxic or harmful gases were produced, except for a small amount of carbon dioxide.

## Figures and Tables

**Table 1:** Laser ablation parameters and their levels.

Manufacturing Parameters	Unit	Notation	Factor Levels				
			1	2	3	4	5
Laser power	W	LP	1.6	2.15	2.7	3.25	3.8
Laser etching speed	mm/s	LES	50	100	150	200	250
Laser ablation times	-	LET	1	3	5	7	9

**Table 2:** The designed L_25_(5^3^) orthogonal experiments.

NO.	A	B	C	LP	LES	LET
1	1	1	1	1.6	50	1
2	1	2	2	1.6	100	3
3	1	3	3	1.6	150	5
4	1	4	4	1.6	200	7
5	1	5	5	1.6	250	9
6	2	1	2	2.15	50	3
7	2	2	3	2.15	100	5
8	2	3	4	2.15	150	7
9	2	4	5	2.15	200	9
10	2	5	1	2.15	250	1
11	3	1	3	2.7	50	5
12	3	2	4	2.7	100	7
13	3	3	5	2.7	150	9
14	3	4	1	2.7	200	1
15	3	5	2	2.7	250	3
16	4	1	4	3.25	50	7
17	4	2	5	3.25	100	9
18	4	3	1	3.25	150	1
19	4	4	2	3.25	200	3
20	4	5	3	3.25	250	5
21	5	1	5	3.8	50	9
22	5	2	1	3.8	100	1
23	5	3	2	3.8	150	3
24	5	4	3	3.8	200	5
25	5	5	4	3.8	250	7

**Table 3:** Experimental results of the L_25_(5^3^) orthogonal experiments.

NO.	LP (W)	LES (mm/s)	LET (-)	W_S_ (μm)	W_H_ (μm)	S_E_ (μm)	Complete Removal
1	1.6	50	1	21.73	36.85	18.32	T
2	1.6	100	3	27.86	41.46	11.13	T
3	1.6	150	5	22.78	37.46	11.11	T
4	1.6	200	7	23.76	37.37	8.83	T
5	1.6	250	9	21.05	37.19	8.67	T
6	2.15	50	3	25.87	41.86	10.57	T
7	2.15	100	5	29.78	39.8	9.55	T
8	2.15	150	7	23.78	41.41	8.67	T
9	2.15	200	9	26.16	39.16	7.9	T
10	2.15	250	1	18.56	30.33	4.6	F
11	2.7	50	5	39.65	44.4	12.12	T
12	2.7	100	7	27.04	40.29	8.42	T
13	2.7	150	9	37.82	45.17	10.93	T
14	2.7	200	1	24.18	29.72	8.3	F
15	2.7	250	3	21.20	36.55	13.26	F
16	3.25	50	7	46.41	54.00	18.3	T
17	3.25	100	9	29.05	45.87	18.18	F
18	3.25	150	1	27.92	42.23	14.73	F
19	3.25	200	3	25.54	36.23	12.96	T
20	3.25	250	5	30.52	36.57	12.2	T
21	3.8	50	9	52.95	72.04	11.49	T
22	3.8	100	1	34.72	45.54	19.58	T
23	3.8	150	3	32.79	38.74	14.22	T
24	3.8	200	5	36.35	37.8	16.45	T
25	3.8	250	7	38.34	39.04	15.38	T

**Table 4:** ANOVA test for W_S_.

Factor	SS	DOF	MS	F	*p*	Contribution Effect %
LP (W)	773.902	4	193.476	8.929	0.001	46.05%
LES (mm/s)	394.576	4	98.644	4.553	0.018	23.48%
LET (-)	252.037	4	63.009	2.908	0.068	15.00%
Residual	260.007	12	21.667			15.47%

**Table 5:** ANOVA test for W_H_.

Factor	SS	DOF	MS	F	*p*	Contribution Effect %
LP (W)	267.754	4	66.938	2.621	0.088	16.80%
LES (mm/s)	652.780	4	163.195	6.389	0.005	40.97%
LET (-)	366.421	4	91.605	3.587	0.038	23.00%
Residual	306.496	12	74.027			19.23%

**Table 6:** ANOVA test for S_E_.

Factor	SS	DOF	MS	F	*p*	Contribution Effect %
LP (W)	191.318	4	47.829	4.870	0.014	52.94%
LES (mm/s)	44.506	4	11.126	1.133	0.387	12.32%
LET (-)	7.697	4	1.924	0.196	0.936	2.13%
Residual	117.859	12	9.822			32.61%

**Table 7:** Range analysis table based on the evaluation of W_S_.

	LP (W)	LES (mm/s)	LET (-)
-K1-K1	23.44	37.32	25.42
-K2-K2	24.83	29.69	26.65
-K3-K3	29.98	29.02	31.82
-K4-K4	31.89	27.20	31.87
-K5-K5	39.03	25.93	33.41
Optimal Level	1	5	1
R_j_	15.59	11.39	7.99
Order of range	R_1_ > R_2_ > R_3_		

**Table 8:** Range analysis table based on the evaluation of W_H_.

	LP (W)	LES (mm/s)	LET (-)
-K1-K1	38.07	49.83	36.93
-K2-K2	38.51	42.59	38.97
-K3-K3	39.23	41.00	39.21
-K4-K4	42.98	36.06	42.42
-K5-K5	46.63	35.94	47.89
Optimal Level	1	5	1
R_j_	8.56	13.89	10.96
Order of range	R_2_ > R_3_ > R_1_		

**Table 9:** Range analysis table based on the evaluation of S_E_.

	LP (W)	LES (mm/s)	LET (-)
-K1-K1	11.61	13.11	13.11
-K2-K2	8.26	12.43	12.43
-K3-K3	10.61	12.29	12.29
-K4-K4	15.27	11.92	11.92
-K5-K5	15.42	11.43	11.43
Optimal Level	2	5	5
R_j_	7.16	1.68	1.68
Order of range	R_1_ > R_2_ > R_3_		

## Data Availability

Data will be made available on request.

## References

[1] Blaschta F., Schulze K., Schulz S.E., Gessner T. (2004). SiO_2_ aerogel ultra low k dielectric patterning using different hard mask concepts and stripping processes. Microelectron. Eng..

[2] Zheng X., Guo F., Liu Y., Hu G., Wang Q., Xu M. (2024). Semi-interpenetrating networks of ANF/SiO_2_ composite aerogel with lightweight, compressible, and excellent flame retardancy properties. J. Non-Cryst. Solids.

[3] Ni H., Wang D., Jia R., Liu Y., Wu D., Chang S., Li X., Shi J. (2025). Fast preparation of aluminum silicate fiber reinforced silica aerogel composites with the ultra-low thermal conductivity and high temperature resistance by combustion technology. J. Alloys Compd..

[4] Fuzhou M., Ruiming Y., Bihui L., Bai Y. (2022). Research Progress of High-Temperature Wave-transparent Ceramic Materials for Radome. Cem. Carbides.

[5] Zhang Y. (2023). Broadband and Wide-Angle RCS Reduction Techniques for Antennas. Master’s Thesis.

[6] Lee K.-F., Tong K.-F. (2012). Microstrip patch antennas—Basic characteristics and some recent advances. Proc. IEEE.

[7] Chang M.-J., Zhu W.-Y., Liu J., Bai G., Li X., Lu X.-Q., Lei Y.-H. (2024). Fabrication of elastic SiO_2_ aerogels with prominent mechanical strength and stability reinforced by SiO_2_ nanofibers and polyurethane for oil adsorption. Sep. Purif. Technol..

[8] Li Z., Zhou F., Hu M., Liu M., Sun M., Chen Z., Wu X., Liu Q. (2024). Enhancing thermal safety of hydrophobic SiO_2_ aerogels through introducing ammonium polyphosphate intercalated layered double hydroxides. Ceram. Int..

[9] Chang M.-J., Bai G., Liu J., Hu Q.-Y., Hu S.-Y., Yin Y., Cao S.-S. (2025). SiO_2_ nanofiber and graphene oxide assisted fabrication of silica aerogels with novel interpenetration networks for efficient oil adsorption and photothermal evaporation. J. Environ. Chem. Eng..

[10] Chae J., Park S.S., Freiheit T. (2006). Investigation of micro-cutting operations. Int. J. Mach. Tools Manuf..

[11] Xue B., Geng Y., Yan Y., Ma G., Wang D., He Y. (2020). Rapid prototyping of microfluidic chip with burr-free PMMA microchannel fabricated by revolving tip-based micro-cutting. J. Mater. Process. Technol..

[12] Cristoforetti A., Gamba M., Brenna A., Ormellese M., Fedel M. (2025). Influence of intermetallics on through-mask electrochemical micromachining for surface texturing of aluminum alloys. Surf. Coat. Technol..

[13] Chen X., Fan G., Saxena K.K., Qian J., Reynaerts D. (2020). Through metallic mask electrochemical micromachining of micro-groove with a porous cathode. Procedia CIRP.

[14] Yunos L., Murphy P.J., Jane M.L., Zuber K. (2025). Frequency selective surface microstructure patterning on silver-based low-e coatings: Laser ablation and photolithography. Appl. Surf. Sci. Adv..

[15] Ma J., Zhang H., Ye T., Wang S., Yang Z.-B., Jia Z. (2024). New method of continuous-wave laser ablation for processing microgroove with variable cross-section. Opt. Laser Technol..

[16] Ji P., Zhang Y. (2017). Melting and thermal ablation of a silver film induced by femtosecond laser heating: A multiscale modeling approach. Appl. Phys. A.

[17] Hwang J.S., Park J.-E., Kim G.W., Lee H., Yang M. (2020). Near-infrared nanosecond pulsed laser ablation of silver nanowire in aqueous media for low-power and low-debris laser processing. J. Micromech. Microeng..

[18] Ostendorf A., Kulik C.J., Temme T., Otte F., Samm K., Miyamoto I., Helvajian H., Itoh K., Kobayashi K.F., Ostendorf A., Sugioka K. (2004). The influence of physical characteristics on ablation effects in UV laser assisted micro-engineering. Proceedings of the SPIE Proceedings.

[19] Ning J., Yu H., Zhao J.-X., Zhang L.-J., Na S.-J. (2025). Thermal damage behavior and material removal mechanism during femtosecond and nanosecond laser ablation of refractory metal molybdenum. J. Mater. Res. Technol..

[20] Jiang K., Zhang P., Song S., Sun T., Chen Y., Shi H., Yan H., Lu Q., Chen G. (2024). A review of ultra-short pulse laser micromachining of wide bandgap semiconductor materials: SiC and GaN. Mater. Sci. Semicond. Process..

[21] Rouhani M., Metla S.B.S., Hobley J., Karnam D., Hung C.-H., Lo Y.-L., Jeng Y.-R. (2025). A complete phase distribution map of the laser affected zone and ablation debris formed by nanosecond laser-cutting of SiC. J. Mater. Process. Technol..

[22] Zubauskas L., Markauskas E., Vyšniauskas A., Stankevič V., Gečys P. (2024). Comparative analysis of microlens array formation in fused silica glass by laser: Femtosecond versus picosecond pulses. J. Sci. Adv. Mater. Devices.

[23] Lauzurica S., Molpeceres C. (2010). Assessment of laser direct-scribing of a-Si:H solar cells with UV nanosecond and picosecond sources. Phys. Procedia.

[24] Olbrich M., Punzel E., Lickschat P., Weißmantel S., Horn A. (2016). Investigation on the Ablation of thin Metal Films with Femtosecond to Picosecond-pulsed Laser Radiation. Phys. Procedia.

[25] Shin S., Lee W., Park J.K. (2024). Wavelength selection for femtosecond laser processing of materials: Comparison of ablation efficiency and surface quality. Opt. Laser Technol..

[26] Zou R., Zhang L.-H., Li Q.-H., Xu G.-J., He Y.-Z., Zhao Y.-Y., Ma R.-D., Jin F., Xu S.-T., Cao H.-Z. (2025). Femtosecond laser induced hybrid additive/subtractive manufacturing for programmable silver micro/nanostructures. Mater. Today Nano.

[27] Lim M., Kim H.-J., Ko E.-H., Choi J., Kim H.-K. (2016). Ultrafast laser-assisted selective removal of self-assembled Ag network electrodes for flexible and transparent film heaters. J. Alloys Compd..

[28] Takabayashi K., Takahashi T., Tsuchiya E., Mimura K., Yamamoto Y., Kobayashi Y., Tomita T., Yamaguchi M. (2022). Morphology and structure of diamond-like carbon film induced by picosecond laser ablation. Appl. Phys. A.

[29] Yüce C., Okamoto K., Karpowich L., Adrian A., Willenbacher N. (2019). Non-volatile free silver paste formulation for front-side metallization of silicon solar cells. Sol. Energy Mater. Sol. Cells.

[30] Waterhouse G.I.N., Bowmaker G.A., Metson J.B. (2002). Interaction of a polycrystalline silver powder with ozone. Surf. Interface Anal..

[31] Waterhouse G.I.N., Bowmaker G.A., Metson J.B. (2001). Oxidation of a polycrystalline silver foil by reaction with ozone. Appl. Surf. Sci..

[32] Girard D., Escamilla A.R., Variola F., Berini P., Weck A. (2025). Ultrafast laser-induced formation of AgO and Ag_2_O on silver. Appl. Surf. Sci..

[33] Barclay A.J., McKellar A.R.W., Moazzen-Ahmadi N. (2021). New infrared spectra of CO_2_–Ne: Fundamental for CO_2_–22Ne isotopologue, intermolecular bend, and symmetry breaking of the intramolecular CO_2_ bend. Chem. Phys. Lett..

[34] He G., Li J., Han H., Yan J., Xie J., Luo M., Zhao Y., Cheng Y., Qiao M. (2025). Exploring of the effects of thermal ablation and plasma expansion on structure formation during ultrafast laser processing of Ti6Al4V alloy. J. Mater. Process. Technol..

[35] Balachninaitė O., Skruibis J., Matijošius A., Vaičaitis V. (2022). Temporal and spatial properties of plasma induced by infrared femtosecond laser pulses in air. Plasma Sources Sci. Technol..

